# The establishment and application of preimplantation genetic haplotyping in embryo diagnosis for reciprocal and Robertsonian translocation carriers

**DOI:** 10.1186/s12920-017-0294-x

**Published:** 2017-10-17

**Authors:** Shuo Zhang, Caixia Lei, Junping Wu, Jing Zhou, Haiyan Sun, Jing Fu, Yijuan Sun, Xiaoxi Sun, Daru Lu, Yueping Zhang

**Affiliations:** 1 Shanghai Ji Ai Genetics & IVF Institute, Obstetrics and Gynecology Hospital, Fudan University, 588 Fangxie Rd, Shanghai, 200011 China; 2 Obstetrics and Gynecology Hospital, Fudan University, 588 Fangxie Rd, Shanghai, 200011 China; 3 Collaborative Innovation Center for Genetics and Development, School of Life Sciences, Fudan University, 2005 Songhu Rd, Shanghai, 200438 China

**Keywords:** Single nucleotide polymorphism, Breakpoint, Preimplantation genetic haplotyping, Reciprocal translocation, Robertsonian translocation

## Abstract

**Background:**

Preimplantation genetic diagnosis (PGD) is now widely used to select embryos free of chromosomal copy number variations (CNV) from chromosome balanced translocation carriers. However, it remains a difficulty to distinguish in embryos between balanced and structurally normal chromosomes efficiently.

**Methods:**

For this purpose, genome wide preimplantation genetic haplotyping (PGH) analysis was utilized based on single nucleotide polymorphism (SNP) microarray. SNPs that are heterozygous in the carrier and, homozygous in the carrier’s partner and carrier’s family member are defined as informative SNPs. The haplotypes including the breakpoint regions, the whole chromosomes involved in the translocation and the corresponding homologous chromosomes are established with these informative SNPs in the couple, reference and embryos. In order to perform this analysis, a reference either a translocation carrier’s family member or one unbalanced embryo is required. The positions of translocation breakpoints are identified by molecular karyotypes of unbalanced embryos. The recombination of breakpoint regions in embryos could be identified.

**Results:**

Eleven translocation families were enrolled. 68 blastocysts were analyzed, in which 42 were unbalanced or aneuploid and the other 26 were balanced or normal chromosomes. Thirteen embryos were transferred back to patients. Prenatal cytogenetic analysis of amniotic fluid cells was performed. The results predicted by PGH and karyotypes were totally consistent.

**Conclusions:**

With the successful clinical application, we demonstrate that PGH was a simple, efficient, and popularized method to distinguish between balanced and structurally normal chromosome embryos.

**Electronic supplementary material:**

The online version of this article (10.1186/s12920-017-0294-x) contains supplementary material, which is available to authorized users.

## Background

Balanced translocation is a relatively common structural chromosome rearrangement that occurs when an exchange of terminal segments happens between different chromosomes, including Reciprocal and Robertsonian translocation [1]. It has an estimated prevalence of 0.19% in the general population and 2.2% in patients who undergo in vitro fertilization (IVF) treatment and experience a history of recurrent miscarriages or repeated IVF failure [2]. Although carriers of balanced chromosomal rearrangements usually have a normal phenotype, the risk of producing unbalanced gametes is high due to complex segregation modes during meiosis [3]. A quadrivalent structure is formed at meiosis I through pairing of translocated chromosomes and the two corresponding normal chromosomes. This structure commonly undergoes one of the three modes of segregation: 2:2, 3:1 or 4:0. Only the alternate segregation pattern in 2:2 mode can result in normal or balanced gametes, the other segregation patterns will generate unbalanced types of gametes which can lead to apparent infertility, recurrent miscarriage, or delivery of a phenotypically abnormal offspring with mental retardation or other congenital abnormalities [4, 5].

For couples with a known balanced chromosome translocation, preimplantation genetic diagnosis (PGD) with fluorescent in-situ hybridization (FISH) has been used to select normal/ balanced embryos for transfer [6, 7]. However, FISH is limited by technical difficulties such as cell fixation and signal interpretation [8]. In recent years, microarray technologies and next-generation sequencing (NGS) which are capable of testing for chromosome translocation imbalances and screening for aneuploidy of all 23-pairs of chromosomes simultaneously have been widely used in many centers [9–12]. Studies performed previously indicate that array-based PGD can improve pregnancy rates in translocation carrier couples to between 45%-70% per transfer [12–14]. Despite this advantage, these traditional PGD methods can’t distinguish balanced and structurally normal embryos from translocation carriers. To date, some feasible technologies have been introduced to solve this difficulty. FISH with specific probes spanning the chromosome breakpoints was used earlier [15, 16]. Treff and colleagues enrolled reciprocal translocation families and predicted the balanced or structurally normal embryos based upon genotype within five Mb of the breakpoints [17]. The unbalanced embryos were used as a reference. In addition, in the latest research of Hu et al. [18], the authors developed a “MicroSeq-PGD” method which combined chromosome microdissection and NGS in reciprocal translocation carriers to characterize the DNA sequence of the translocation breakpoints to distinguish embryos. However, these methods are possibly time-consuming, complicated, and homologous recombination can’t be identified. More importantly, Robertsonian translocation carriers are not included, which have an estimated 0.1% incidence rate in the general population [19].

Therefore, the most suitable methodological design for translocation carriers has not been well established. Preimplantation genetic haplotyping (PGH) was first introduced for preimplantation genetic testing of single gene defects by polymorphic short tandem repeat (STR) markers [20]. In this current study, our aim was to utilize PGH to distinguish between balanced and structurally normal embryos prior to implantation from both reciprocal translocation and Robertsonian translocation carriers accurately, along with the genetic screening for all 23-pairs of chromosomes. For this purpose, a SNP microarray was used [21]. Haplotypes including the breakpoint regions, the whole chromosomes involved in the translocation and corresponding normal homologous chromosomes were established using informative SNP markers. The carrier’s family member or an unbalanced embryo was used as a reference. The laboratory technicians were blinded regarding the PGH results. The predictive accuracy of PGH was validated by the conventional amniotic fluid karyotypes in the second trimester.

## Methods

### Patients

Eleven translocation carrier families that would undergo assisted reproductive were enrolled in Shanghai Ji Ai Genetics & IVF Institute from June 2014 to Marth 2016. All families had a history of a recurrent spontaneous abortion, infertility or pregnancies with chromosome anomalies. The translocation karyotypes were 46,XY, t(5;22) (q33; q12); 46,XX,t (16; 18) (q22;q21.1); 46,XX,t(12;22)(p12;q13); 46,XX,t(11;16) (p11.2; p13.1); 46, XY, t (1;19) (q12; p13); 45,XX, rob(14;21) (q10;q10); 45,XY, rob(14; 21) (q10; q10); 45, XX, rob (14; 15) (q10;q10); 46,XY,t(6;9)(q27;q22); 46,XX,t(2;3) (q22.1; p14.1); 46,XY, t(7; 11) (q21;q21), respectively. Ten ml peripheral blood from each couple and family members was collected at recruitment. Written informed consent was obtained from each family and the study protocol was approved by the Ethics Committee for Human Subject research of the Obstetrics and Gynecology Hospital, Fudan University.

### Blastocyst biopsy and WGA

For embryos at the blastocyst stage, three to ten cells were removed from the trophectoderm on day five or six of embryonic development. The biopsied cells were placed into polymerase chain reaction tubes with an alkaline denaturation buffer for cell lysis as previously describe. Whole genomic amplification (WGA) was performed by the multiple displacement amplification (MDA) method. Isothermal DNA amplification with phi 29 DNA polymerase was performed (Repli-g single cell kit, QIAGEN GmbH, Hilden, Germany) as described in the manufacturers’ protocol. The isothermal amplification was performed at 30 °C for 8 h and the reaction was stopped by incubation at 65 °C for 3 min.

### SNP-array and analysis

SNP genotypes were performed with Illumina Human Karyomap-12 V1.0 microarray in this study as previously described [21]. Each Karyomap-12 bead chip contained approximately 300,000 SNPs. Molecular karyotypes and haplotypes could be established with this method simultaneously in each embryo. This information was then used to identify the normal embryos free of chromosomal copy number variations (CNV) and distinguish between balanced and structurally normal embryos respectively. The molecular karyotype analysis and the linkage analysis of haplotype were performed with Bluefuse®-Multi software (Illumina, Inc. San Diego, USA).

Informative SNPs were used to establish the haplotypes including the breakpoint regions, the whole chromosomes involved in the translocation and the corresponding normal homologous chromosomes in the couple, reference and embryos. The informative SNPs of ±2 Mb around the breakpoints were selected to establish haplotypes of the regions covering the breakpoints. The selection criteria for informative SNPs, was that they should be heterozygous in the carrier and homozygous in his/her partner. Also these SNPs should be homozygous in the carrier’s parents or other family members. This information was used to determine which of the carrier’s two haplotypes were linked to the derivative chromosome or to the normal chromosome. The carrier’s family member or an unbalanced embryo was used as a reference. If the carrier’s unbalanced embryo was used as a reference, then there was no requirement to study family members.

The haplotypes of the whole chromosomes involved in the balanced translocation and the corresponding normal homologous chromosomes could indicate the presence of homologous recombination around the breakpoints. The predictive criterion to distinguish balanced and structurally normal embryos combined the haplotypes of breakpoint regions and the presence of homologous recombination in these regions. When recombination doesn’t occur, if the embryo carries the same haplotype with the carrier’s family member who has the same translocation or unbalanced embryos, or if the embryo carries the different haplotype with the carrier’s family member who has the normal karyotype, and therefore it will be defined as a balanced translocation embryo; If the embryo carries the same haplotype with the carrier’s family member who has the normal karyotype, or if the embryo carries the different haplotype with the carrier’s family member who has the same translocation or unbalanced embryos, then it will be defined as a structurally normal embryo. When recombination occurs, the result might be hard to predict due to the complexity. The consistency of predictive results with different reference samples including family members and unbalanced embryos was compared. The predictive accuracy of PGH was validated by a blinded comparison with conventional amniotic fluid cell karyotypes in the second trimester of successful pregnancies after embryo transfer.

## Results

In this study, the 11 balanced translocation families underwent 14 IVF cycles. Family10 underwent three cycles, and the other families had one cycle each. The characteristics and ovarian stimulation results of these patients are listed in Table 1. In families 1-8, the translocation was inherited from a parent. In family 9, the carrier’s parents had already died and the couple didn’t have unbalanced embryos, but the carrier’s sister and brother were both identified to carry the same translocation. In family 10 and 11, the two couples didn’t tell their parents that they were undergoing IVF treatment and the carrier was an only child, therefore we couldn’t get the peripheral blood karyotype of their family members. While they expressed a strong desire to transfer the structurally normal embryo, therefore the unbalanced embryos were used as reference. With our method, we obtained molecular karyotypes from all the 68 biopsied blastocysts. Of the 68 diagnosed blastocysts, 26 were balanced or normal, 25 blastocysts had translocation related abnormalities and 17 blastocysts showed de novo abnormalities unrelated to the translocation. PGH analysis then was performed in the 26 blastocysts that were balanced or normal, which indicated that 12 were balanced and 14 were structurally normal embryos. The predictive results of two different breakpoint regions in each embryo were consistent with the amniotic fluid analysis in ongoing pregnancies.

**Table 1:** The characteristics of the patients in this study

Family	Maternal age /Paternal age	Karyotype^a^	Reason for karyotyping	The number of oocytes retrievedf	The number of mature oocytes (MII)	The number of fertilized oocytes	The number of D3 oocytes	The number of biopsied blastocysts
1	33/34	46,XY,t(5;22)(q33;q12) mat	Infertility	16	15	15	15	5
2	33/30	46,XX,t(16;18) (q22; q21.1) pat	repeated miscarriage	21	16	15	11	4
3	23/31	46,XX,t(12;22)(p12;q13) pat	repeated miscarriage	14	8	5	5	3
4	29/31	46,XX,t(11;16)(p11.2;p12.3) pat	affected fetus	15	13	9	9	6
5	29/29	46,XY,t(1; 19) (q12;p13) mat	repeated miscarriage	19	14	12	11	6
6	27/26	45,XX, rob(14; 21) (q10;q10) mat	repeated miscarriage	18	12	7	7	6
7	31/33	45,XY, rob(14; 21) (q10;q10) pat	Infertility	16	16	16	13	8
8	26/29	45,XX, rob(14; 15) (q10;q10) pat	repeated miscarriage	4	3	3	3	3
9	36/53	46,XY,t(6;9)(q27;q22) mat/pat	repeated miscarriage	8	8	5	5	3
10^b^	29/30	46,XX, t(2;3)(q22.1;p14.1)	repeated miscarriage	31	26	26	21	12
11	25/28	46,XY, t(7;11)(q21;q21)	Infertility	28	28	26	22	12

^a^The karyotypes were identified by peripheral blood cells

^b^In family-10, the number of oocytes was from 3 cycles

**Fig. 1:**
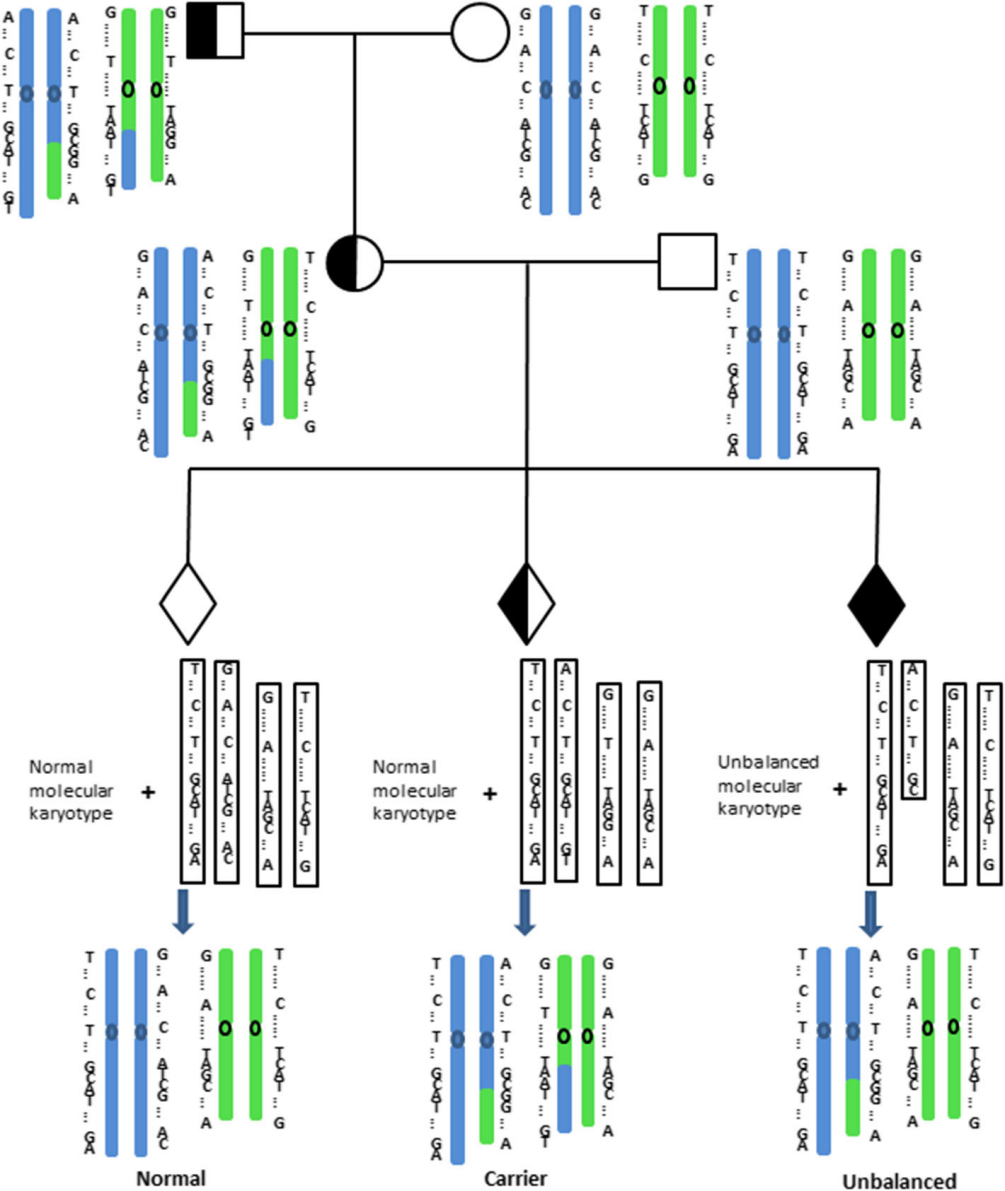
The process of establishing haplotypes and distinguishing between balanced and structurally normal chromosomes embryos through PGH analysis. Informative SNPs should be heterozygous in the carrier, and homozygous in the carrier’s partner and carrier’s family member. These SNPs were used to establish the haplotypes of the breakpoint regions, the whole chromosomes involved in the translocation and the corresponding normal homologous chromosomes in the couple, reference and embryos

**Fig. 2:**
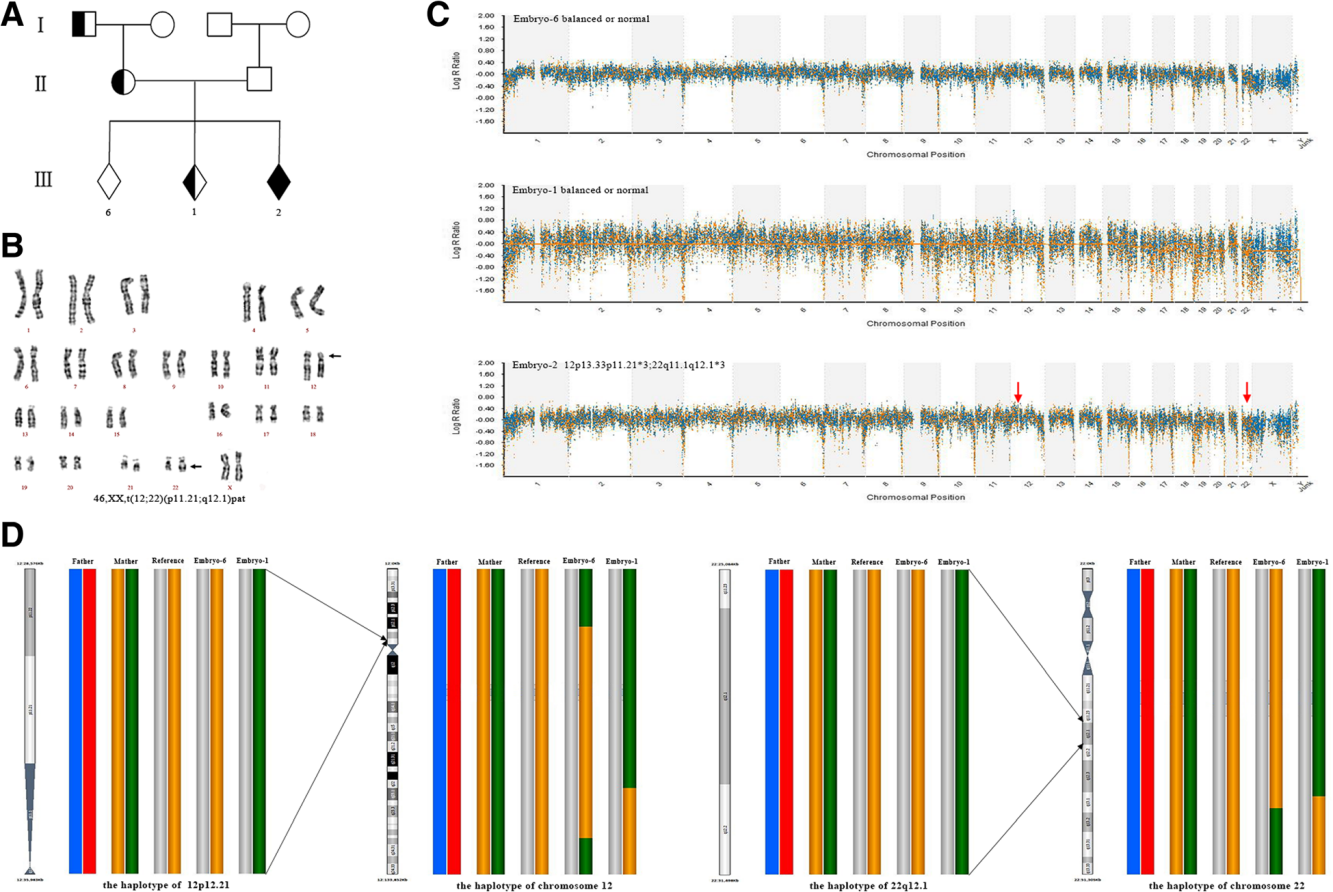
**a** The genealogic tree in family3. **b** The peripheral blood karyotype of carrier, the translocation was inherited from her father. **c** Based on the genetic screening of 23-pairs chromosomes, embryo-1 and embryo-6 were identified as balanced or normal embryos, embryo-2 was identified as unbalanced embryo. **d** The haplotypes including the two breakpoint regions, the two whole chromosomes involved in the translocation and the two corresponding normal homologous chromosomes in the couple, reference who has the same translocation and embryos were shown. The recombination was identified outside the breakpoints. The colorful histograms represented haplotypes, in the embryos the gray column represented the haplotype that was inherited from the normal parent and in the carrier’s family number the gray column represented the haplotype that wasn’t passed on to the carrier, the other different colorful histograms represented different haplotypes. The PGH result indicated embryo-6 was a translocation carrier embryo and embryo-1 was a structurally normal embryo

Haplotypes were assigned for the carrier, the partner, the embryos and the carrier’s family member. The molecular karyotypes of unbalanced embryos could help to pinpoint the relatively accurate position of breakpoint. If the breakpoint couldn’t be identified by unbalanced embryos, then that from the peripheral blood karyotype was used. Actually, the haplotype of any region or chromosome genome wide could be established and therefore this method is universal for any kind of translocation. Detailed results of the microarray platform of the transferred blastocysts were shown in Table 2, the other non- transferred embryos are in Additional file 1: Table S1.The process of establishing haplotypes and distinguishing between balanced and structurally normal chromosome embryos through PGH analysis was shown in Fig. 1. The haplotypes of two breakpoint regions and the chromosomes involved in the translocation and the normal homologous chromosomes in family 3 are shown in Fig. 2. The summary of informative SNPs that were used to establish the whole haplotypes of the successfully transferred blastocysts is listed in Table 3, the other non- transferred embryos are in Additional file 2: Table S2. In family 2, 5, 6 and 7, the predictive results using family members or unbalanced embryos with deletion were consistent, which was shown in Table 2. The other families had either no unbalanced embryos with deletion or no available family members, then the comparison of predictive results couldn’t be performed.

**Table 2:** Detailed results of microarray platform of the transferred blastocysts

Family	The number of biopsied blastocysts	Grade of blastocysts	Molecular karyotype	Results of PGH		Transferred blastocysts	Karyotype of amniotic fluid	Consistency?
				Family member^a^	Unbalanced embryos^b^			
1	7	6BB	(1-22,X)*2	Carrier	NA	Embryo-1	46,XX,t(5;22)(q33;q12)	Yes
2	4	5 BC	(1-22,X)*2	Normal	Normal	Embryo-4	46,XX	Yes
3	3	5BB	(1-22)*2, (XY)*1	Carrier	NA	Embryo-6	46,XY,t(12;22)(p12;q13)	Yes
4	6	5BB	(1-22,X)*2	Normal	NA	Embryo-1	46,XX	Yes
5	6	5AB	(1-22,X)*2	Carrier	Carrier	Embryo-1	46,XX,t(1; 19) (q12;p13)	Yes
6	6	5 BC	(1-22,X)*2	Carrier	Carrier	Embryo-9	45,XX, rob(14; 21)(q10;q10)	Yes
7	8	5 BC	(1-22,X)*2	Normal	Normal	Embryo-6	46,XX	Yes
8	3	5BB	(1-22)*2, (XY)*1	Carrier	NA	Embryo-2	45,XY,der(14;15)(q10;q10)	Yes
9	3	5AB	(1-22,X)*2	Normal	NA	Embryo-4	46,XX	Yes
10	12	5 AC	(1-22,X)*2	NA	Carrier	Embryo-1-5	No pregnancy	NA
		5BB	(1-22,X)*2	NA	Carrier	Embryo-3-4	No pregnancy	NA
		5 BC	(1-22)*2, (XY)*1	NA	Normal	Embryo-3-1	46,XY	Yes
11	12	5 BC	(1-22,X)*2	NA	Normal	Embryo-12	46,XX	Yes

NA = not available

^a^In family1-9, the family member was used as a reference; in family10-11, the unbalanced embryo was used as a reference

^b^In family2, embryo-1, embryo-4 and embryo-9 were included; In family5, embryo-3 was included; In family6, embryo-4 was included; In family7, embryo-13 was included; In other families, the unbalanced embryos couldn’t be used as reference

**Table 3:** Summary of informative SNPs used to establish the whole haplotypes of the successfully transferred blastocysts

Family^a^	Number of blastocysts	Chromosome	The total number of informative SNPs	The average number of informative SNPs /Mb	The number of recombination SNPs	The location of recombination	The location of breakpoint^b^	Weather recombination occurs in the breakpoint?
1	Embryo-1	5	1520	8.4	91	5:1-7,228,178(p15.33p15.31)	5q33.1	No
		22	401	7.8	0	NR	22q12	No
2	Embryo-4	16	823	9.1	148	16:1-9,724,564(p13.3p13.2)	16q23.1	No
		18	637	8.2	197	18:7,105,507-31,960,623(p11.23q12.1)	18q21.31	No
3	Embryo-6	12	1121	8.4	218	12:1-25,329,895(p13.33p12.1)	12p11.21	No
					163	12:117,913,186-133,851,895(q24.22q24.33)		No
		22	419	8.2	149	22:40,212,715-51,304,566(q13.1q13.33)	22q12.1	No
4	Embryo-1	11	1328	9.8	309	11:94,616,073-127,991,048(q21q24.3)	11p11.2	No
		16	648	7.2	209	16:1-16,599,806(p13.3p13.11)	16p12.3	No
5	Embryo-1	1	1802	7.2	763	1:15,110,819-116,270,101(p36.21p13.2)	1q21.2	No
					117	1:235,510,141-249,250,261(q42.3q44)		No
		19	482	8.2	115	19:51,465,236-59,128,983(q13.41q13.43)	19p13.11	No
6	Embryo-9	14	672	6.3	0	NR	Centromere	No
		21	350	7.3	0	NR	Centromere	No
7	Embryo-6	14	776	7.2	558	14:44,534,715-107,349,540(q21.2q32.33)	Centromere	No
		21	363	7.5	0	NR	Centromere	No
8	Embryo-2	14	787	7.3	192	14:65,512,354-89,493,151(q23.3q31.3)	Centromere	No
		15	785	7.7	0	NR	Centromere	No
9	Embryo-4^c^	6	1565	9.2	197	6:37,549,903-56,107,530(p21.2p12.1)	6q27	No
					58	6:60,539,141-65,772,043(q11.1q12)		No
					19	6:85,870,071-88,787,897(q14.3q15)		No
					40	6:168,092,007-171,115,067(q27)		No
		9	936	6.7	9	9:2,331,347-2,792,531(p24.3p24.2)		No
					424	9:5,098,509-77,784,684(p24.1q21.13)	9q22	No
	Embryo-4^d^	6	751	4.4	13	6:11,558,464-14,305,025(p24.2p23)	6q27	No
					197	6:37,549,903-56,107,530(p21.2p12.1)		No
					57	6:60,539,141-65,772,043(p11.1q12)		No
		9	367	2.6	179	9:12,809,535-76,736,238(p23q21.13)	9q22	NA^e^

NR = no recombination; NA = not available

^a^In family10-11, as the unbalanced embryos were the only reference, the whole chromosome haplotypes couldn’t be established

^b^The breakpoints were identified by microarray results, except chromosome 22 in family1

^c^The carrier’s brother was used as a reference

^d^The carrier’s sister was used as a reference

^e^The haplotype couldn’t be established in this breakpoint region, for no informative SNPs existed

When finishing the PGH analysis, part of the families had already had their embryos transferred. Fourteen blastocysts were thawed and the transfer cycles were performed. In family 10, the women failed to achieve pregnancy in the first and second transfer cycle, while she was successful in the third cycle. In the other families, all became pregnant in the first cycle. For all the women that were pregnant after embryo transfer, cytogenetic analysis of amniotic fluid was required to be performed in the second trimesters (Table 2). We proved the predictive results of PGH and cytogenetic results of amniotic fluid cells were totally consistent. The sensitivity and specificity were 100%, respectively.

## Discussion

Despite having a successful PGD cycle and delivery in translocation families, many of these couples will be passing on the translocation to their children who may also be subjected to infertility, recurrent pregnancy loss or even have to seek assisted reproductive technologies to finally conceive. In our center, many couples express a strong desire to pursue more careful screening and transfer structurally normal embryos, especially for carriers with an abnormal phenotype. It is known that about 6% of translocation carriers present with a series of symptoms such as autism, mental retardation, or congenital abnormalities [22, 23].

Many researchers have attempted to overcome the difficulty in determining which embryos are non-carriers of a familial translocation. Initially, FISH with chromosome specific probes spanning the translocation breakpoints were used to differentiate between normal or balanced embryos [15, 16]. Although relatively feasible, the techniques used were extremely complicated and not suitable for routine clinical diagnosis. Treff and colleagues enrolled reciprocal translocation families and predicted the balanced or normal status of each embryo based upon SNP genotypes within five Mb of the breakpoints using the Affymetrix NspI Gene Chip [17], in these cases the unbalanced embryos were the only reference used. In addition, in the latest research of Hu et al. [18], the authors developed the “MicroSeq-PGD” method which combined chromosome microdissection technique and NGS, to distinguish between balanced and structurally normal embryos in reciprocal translocation carriers by junction spanning PCR sequencing analysis and/or linkage analysis. This method could identify the translocation breakpoints and find the disrupted genes precisely. However, these current methods are time-consuming, complicated, and not available in most reproductive centers. More importantly, Robertsonian translocation carriers were not included, as the authors couldn’t find the breakpoints which are located in the centromeric regions. Moreover, homologous recombination couldn’t be identified using these methods.

Compared with these studies, several obvious advantages could be concluded in our research. First, the prediction for the status of chromosomes in embryos and the genetic screening for 23-pairs of chromosomes could be performed simultaneously using our method. Second, besides the haplotypes of the breakpoint regions, the haplotypes of the two whole chromosomes involved in the translocation and the two corresponding normal homologous chromosomes could be established in the carrier simultaneously, which could show the presence of homologous recombination in the breakpoint regions. Therefore, the prediction of PGH should combine the haplotypes of breakpoint regions and the whole chromosomes. Coincidentally, in this research, we didn’t identify recombination in the breakpoint regions in all the embryos. Third, the haplotype of any region or chromosome genome wide could be established, so our method was universal for any kind of translocation. Fourth, the methodology was relatively simple, including the experiment and date analysis, which was suitable for routine clinical work. The total process could be finished within two days. Fifth, chromosome translocation including reciprocal translocation and Robertsonian translocation, both these two types were able to be analyzed by our method.

Although the predictive results of unbalanced embryos were consistent with the family number in our study, we thought that not all the unbalanced embryos could be used as a reference (such as the unbalanced embryo #5 and embryo #13 in family 1). Here three copies in 5q33.1q35.3 were identified by molecular karyotyping and three haplotypes might potentially exist. It was therefore difficult to determine which of the carrier’s haplotypes are linked to the derivative or to the normal chromosomes. Possibly, only the embryos with deletion of the translocation related fragments could be used. We analyzed semen from 9 male translation carriers with FISH and we found this kind of unbalanced sperm that contained only duplications of translocation related fragments accounted for 0.2% ~ 5.0% of the semen, while an abnormality was not identified in other 18 non- translation carriers semen, possibly the actual situation of meiosis in translocation carriers was much more complex than theoretically [24]. Besides, the unbalanced embryos couldn’t help establish the haplotypes of whole chromosome, and therefore couldn’t indicate whether there was recombination around the breakpoints. To some extent, the haplotype of the whole chromosome was even more important than that of breakpoint region in our opinion, for the former included the latter.

Meanwhile, we found, when the carrier’s sister was use as reference in family-9, near 84 Mb in chromosome 6 (72440951-156,427,957) and 55 Mb in chromosome 9 (85788944- 140,962,305) were same with the carrier besides the derivative chromosomes, therefore the haplotypes of these regions couldn’t be established for lacking of informative SNPs. This might explain the small number of informative SNPs. It’s supposed only half of the carrier’s brothers and sisters could be used as reference although with the same translocation, theoretically the probability was 50% to inherit the different homologous chromosome except the derivative chromosome. Moreover, the called SNPs in microarray from biopsied cell DNA should be less than those from peripheral blood DNA. Overall, for the inherited families, the family member especially carrier’s parents should be the preferred reference; for the de novo families, the unbalanced embryo would be also the choice. The complementarity of the two references might be the best option in clinical, which would be available for more translocation families undergoing IVF treatment. In the method of Hu and colleagues, although the authors didn’t need a reference which was really exciting, two limitations were potential. First, the complexed technology was difficult to be widely popularized in clinical laboratories. Factually, identifying the translocation breakpoints precisely by microdissection was not necessary to predict the chromosome status in embryos. Second, Robertsonian translocation carriers which accounted for 24.3% of all translocation carriers in our center could not use this method.

In addition, we found the average rate of homologous recombination in derivative chromosomes had no differences with the normal chromosomes according to our results [21], meaning that the existing of the quadrivalent structure didn’t reduce the chances of recombination between the paired translocated chromosomes and the two corresponding normal chromosomes. In each Mb distance, 6.6 ± 1.4 SNPs could be used to establish haplotypes, the recombination less than 1 Mb also could be identified in the method.

## Conclusions

In summary, with the validation and successful clinical application in our study, we proved that PGH is an efficient method to distinguish between balanced and structurally normal chromosome embryos from reciprocal and Robertsonian translocation carriers. This study has great clinical signification for these patients. More balanced translocation families would benefit from stopping the passing on of the translocation to their next generation. However, the sensitivity and specificity should be further validated in a larger sample size. Furthermore, PGH should also be used to distinguish between normal and inversion embryos from chromosome inversion carriers.

## Additional files

Additional file 1: Table S1. Detailed results of microarray platform of non-transferred blastocysts. (DOC 114 kb)

Additional file 2: Table S2. Summary of informative SNPs used to establish the whole haplotypes of the non-transferred blastocysts. (DOC 86 kb)

## Abbreviations

CNV: Copy number variation
FISH: Fluorescent in-situ hybridization
IVF: In vitro fertilization
MDA: Multiple displacement amplification
PGD: Preimplantation genetic diagnosis
PGH: Preimplantation genetic haplotyping
SNP: Single nucleotide polymorphism
WGA: Whole genomic amplification
