# Supplementary material for: The establishment and application of preimplantation genetic haplotyping in embryo diagnosis for reciprocal and Robertsonian translocation carriers
Source: BMC Med Genomics. 2017 Oct 17;10:60. doi: 10.1186/s12920-017-0294-x (PMC5646120; doi:10.1186/s12920-017-0294-x)
Supplement: Supplementary file 1 — Detailed results of microarray platform of non-transferred blastocysts. (DOC 114 kb) [file 12920_2017_294_MOESM1_ESM.doc]

| **Additional file 1: Table S1: Detailed results of microarray platform of non-transferred blastocysts** | | | | | | | | | | |  |
| --- | --- | --- | --- | --- | --- | --- | --- | --- | --- | --- | --- |
| Family | | Number of | | Grade of | | | Molecular | | Results of PGH | | |
|  | biopsied blastocysts | | blastocysts | | | karyotype | | Family number**^a^** | | Unbalanced embryos**^b^** | |
| **1** | Embryo-7 | | 5BB | | | (1-22,X)*2 | | Normal | | NA | |
|  | Embryo-5 | | 6BB | | | 5q33.1q35.3*3 | | -- | | -- | |
|  | Embryo-13 | | 5BB | | | 5q33.1q35.3*3 | | -- | | -- | |
|  | Embryo-15 | | 5BB | | | (1-22,X)*2 | | Normal | | NA | |
| **2** | Embryo-1 | | 5BB | | | 16q23.1q24.3*1;18q21.31q23*3 | | -- | | -- | |
|  | Embryo-9 | | 5BB | | | 16q23.1q24.3*3;18q21.31q23*1 | | -- | | -- | |
|  | Embryo-10 | | 5BB | | | 16q23.1q24.3*3;18q21.31q23*1 | | -- | | -- | |
| **3** | Embryo-1 | | 5BC | | | (1-22)*2, (XY)*1 | | Normal | | NA | |
|  | Embryo-2 | | 5BC | | | 12p13.33p11.21*3;22q11.1q12.1*3 | | -- | | -- | |
| **4** | Embryo-2 | | 5BB | | | 2q12.3q37.3*1 | | -- | | -- | |
|  | Embryo-3 | | 5BC | | | 6p12.3q27*1;6p25.3p21.1*3; 11*1; | | -- | | -- | |
|  | Embryo-7 | | 5BC | | | 11p15.5p11.2*3;16p13.3p12.3*3 | | -- | | -- | |
|  | Embryo-8 | | 5CB | | | 16*3 | | -- | | -- | |
|  | Embryo-12 | | 5BC | | | 3*3 | | -- | | -- | |
| **5** | Embryo-2 | | 5BC | | | (1-22)*2, (XY)*1 | | Carrier | | Carrier | |
|  | Embryo-3 | | 5BC | | | 1q21.2q44*1,19p13.3p13.11*3 | |  | |  | |
|  | Embryo-7 | | 5BB | | | (1-22)*2, (XY)*1 | | Carrier | | Carrier | |
|  | Embryo-10 | | 5BB | | | (1-22,X)*2 | | Normal | | Normal | |
|  | Embryo-11 | | 5BC | | | (1-22,X)*2 | | Normal | | Normal | |
| **6** | Embryo-1 | | 5BC | | | (1-22)*2, (XY)*1 | | Normal | | Normal | |
|  | Embryo-3 | | 5BC | | | (1-22)*2, (XY)*1 | | Normal | | Normal | |
|  | Embryo-4 | | 5BC | | | 21*3 | | -- | | -- | |
|  | Embryo-6 | | 5BC | | | X*2,Y*1 | | -- | | -- | |
|  | Embryo-7 | | 5BC | | | 16*3 | | -- | | -- | |
| **7** | Embryo-1 | | 5BC | | | 4*3 | | -- | | -- | |
|  | Embryo-2 | | 5BC | | | 13*1 | | -- | | -- | |
|  | Embryo-9 | | 5BC | | | 16*3 | | -- | | -- | |
|  | Embryo-10 | | 5BB | | | (1-22)*2, (XY)*1 | | Carrier | | Carrier | |
|  | Embryo-12 | | 5BC | | | 1*3 | | -- | | -- | |
|  | Embryo-13 | | 5BB | | | 21*1 | | -- | | -- | |
|  | Embryo-16 | | 5BB | | | (1-22,X)*2 | | Carrier | | Carrier | |
| **8** | Embryo-1 | | 5BC | | | 6*3 | | -- | | -- | |
|  | Embryo-3 | | 5BB | | | 12*1;17*1 | | -- | | -- | |
| **9** | Embryo-2 | | 5BB | | | 22*1 mosaic | | -- | | -- | |
|  | Embryo-5 | | 5BC | | | (1-22)*2, (XY)*1 | | Normal | | NA | |
| **10** | Embryo-1-1 | | | | 5AC | 3p26.3q21.3*3,3q21.3q29*1,15*1 | | -- | | -- | |
|  | Embryo-1-8 | | | | 5BC | 2p25.3q21.1*1,3p14.1q29*3 | | -- | | -- | |
|  | Embryo-2-1 | | | | 5BC | 3*1 | | -- | | -- | |
|  | Embryo-2-2 | | | | 5BC | (1-22,X)*2 | | NA | | Carrier | |
|  | Embryo-2-4 | | | | 5BC | 2p25.3q22.1*1;3p14.1q29*3 | | -- | | -- | |
|  | Embryo-3-2 | | | | 5BC | 3p26.3q21.3*3,3q21.3q29*1,15*1 | | -- | | -- | |
| **10** | Embryo-3-5 | | | | 5BC | 2p25.3q22.1*3;3p14.1q29*1 | | -- | | -- | |
|  | Embryo-3-6 | | | | 5BC | 2p25.3q22.1*1;3p14.1q29*3 | | -- | | -- | |
|  | Embryo-3-8 | | | | 5BC | 2q22.1q37.3*1;3p26.3p14.1*3 | | -- | | -- | |
| **11** | Embryo-5 | | | | 5BC | 7q21.3q36.3*3;11q21q25*1 | | -- | | -- | |
|  | Embryo-7 | | | | 5BB | 7q21.3q36.3*3;11q21q25*1 | | -- | | -- | |
|  | Embryo-9 | | | | 5BC | 18*3 | | -- | | -- | |
|  | Embryo-10 | | | | 5BC | 7q21.3q36.3*1;11q21q25*3 | | -- | | -- | |
|  | Embryo-17 | | | | 5BC | 7q21.3q36.3*3;11q21q25*1 | | -- | | -- | |
|  | Embryo-18 | | | | 5BC | UPD(1-22,X)*2 | | -- | | -- | |
|  | Embryo-19 | | | | 5BC | 4*1 | | -- | | -- | |
|  | Embryo-23 | | | | 5BC | 7q21.3q36.3*1;11q21q25*3 | | -- | | -- | |
|  | Embryo-25 | | | | 5BB | UPD(1-22,X)*2 | | -- | | -- | |
|  | Embryo-26 | | | | 5BC | UPD(1-22,X)*2 | | -- | | -- | |
|  | Embryo-28 | | | | 5BB | 7q21.3q36.3*3;11q21q25*1;20*1 | | -- | | -- | |
| NA= not available | | | | | | | | | | | |
| ^a^ In family1-9, the family number was used as a reference; in family10-11, the unbalanced embryo was used as a reference. | | | | | | | | | | | |
| ^b^ In family2, embryo-1, embryo-4 and embryo-9 were included; In family5, embryo-3 was included; In family6, embryo-4 was included; In family7, embryo-13 was included; In other families, the unbalanced embryos couldn’t be used as reference. | | | | | | | | | | | |
