# Supplementary material for: The establishment and application of preimplantation genetic haplotyping in embryo diagnosis for reciprocal and Robertsonian translocation carriers
Source: BMC Med Genomics. 2017 Oct 17;10:60. doi: 10.1186/s12920-017-0294-x (PMC5646120; doi:10.1186/s12920-017-0294-x)
Supplement: Supplementary file 2 — Summary of informative SNPs used to establish the whole haplotypes of the non-transferred blastocysts. (DOC 86 kb) [file 12920_2017_294_MOESM2_ESM.doc]

| **Additional file 2: Table2. Summary of informative SNPs used to establish the whole haplotypes of the non-transferred blastocysts.** | | | | | | | | |
| --- | --- | --- | --- | --- | --- | --- | --- | --- |
| Family^a^ | Number of | Chromosome | The total number of | The average number of | The number of | The location of | The location of | Weather recombination |
|  | blastocysts |  | informative SNPs | informative SNPs /Mb | recombination SNPs | recombination | breakpoint ^b^ | occurs in the breakpoint? |
| **1** | Embryo-7 | 5 | 1216 | 6.8 | 95 | 5:572663-8672942(p15.33p15.31) | 5q33.1 | No |
|  |  | 22 | 351 | 6.9 | 84 | 22:43926364-51162059(q13.2q13.33) | 22q12 | No |
|  | Embryo-15 | 5 | 1171 | 6.5 | 577 | 5:572663-82201161(p15.33q14.2) | 5q33.1 | No |
|  |  |  |  |  | 48 | 5:176784512-180120528(q35.3) |  | No |
|  |  | 22 | 221 | 4.3 | 0 | NR | 22q12 | No |
| **3** | Embryo-1 | 12 | 914 | 6.8 | 294 | 12:96089862-133851895(q22q24.33) | 12p11.21 | No |
|  |  | 22 | 311 | 6.1 | 110 | 22:38253109-51304566(q13.1q13.33) | 22q12.1 | No |
| **5** | Embryo-2 | 1 | 1539 | 6.2 | 115 | 1:1521595-12073785(p36.33p36.22) | 1q21.2 | No |
|  |  |  |  |  | 361 | 1:203440733-249081330(q32.1q44) |  | No |
|  |  | 19 | 381 | 6.5 | 8 | 19:293934-1917925(p13.3) | 19p13.11 | No |
|  |  |  |  |  | 57 | 19:54810370-58866434(q13.42q13.43) |  | No |
|  | Embryo-7 | 1 | 1500 | 6.0 | 7 | 1:1521595-1939826(p36.33) | 1q21.2 | No |
|  |  |  |  |  | 4 | 1:246505486-247415879(q44) |  | No |
|  |  | 19 | 384 | 6.5 | 7 | 19:58766874-59048311(q13.43) | 19p13.11 | No |
|  | Embryo-10 | 1 | 1305 | 5.2 | 547 | 1:1712230-80854470(p36.33p31.1) | 1q21.2 | No |
|  |  |  |  |  | 22 | 1:244351608-249081330(q44) |  | No |
|  |  | 19 | 315 | 5.3 | 12 | 19:419407-1917925(p13.3) | 19p13.11 | No |
|  |  |  |  |  | 49 | 19:56376574-59048311(q13.43) |  | No |
|  | Embryo-11 | 1 | 1475 | 5.9 | 274 | 1:158058109-207414732(q23.1q32.1) | 1q21.2 | No |
|  |  | 19 | 370 | 6.3 | 52 | 19:56659742-59059729(q13.43) | 19p13.11 | No |
| **6** | Embryo-3 | 14 | 439 | 4.1 | 0 | NR | Centromere | No |
|  |  | 21 | 267 | 5.6 | 125 | 21:35773931-47514219(q22.12q22.3) | Centromere | No |
|  | Embryo-1 | 14 | 514 | 4.8 | 0 | NR | Centromere | No |
|  |  | 21 | 246 | 5.1 | 0 | NR | Centromere | No |
| **7** | Embryo-10 | 14 | 663 | 6.2 | 19 | 14:103202717-106004323(q32.32q32.33) | Centromere | No |
|  |  | 21 | 279 | 5.8 | 66 | 21:38433519-47989804(q22.13q22.3) | Centromere | No |
|  | Embryo-16 | 14 | 687 | 6.4 | 0 | NR | Centromere | No |
|  |  | 21 | 288 | 6.0 | 21 | 21:43442836-48055377(q22.3) | Centromere | No |
| **9** | Embryo-5^c^ | 6 | 1078 | 6.3 | 31 | 6:2787925-6129217(p25.2p25.1) | 6q27 | No |
|  |  |  |  |  | 362 | 6:19922354-65460308(p22.3q12) |  | No |
|  |  |  |  |  | 29 | 6:72567430-77803487(q13q14.1) |  | No |
|  |  |  |  |  | 26 | 6:86049040-91105896(q14.3q15) |  | No |
|  |  |  |  |  | 123 | 6:102033541-123829676(q16.3q22.3) |  | No |
|  |  | 9 | 776 | 5.5 | 325 | 9:2342136-76389514(p24.2q21.13) | 9q22 | No |
|  |  |  |  |  | 229 | 103480387-133305614(q31.1q34.3) |  | No |

NR= no recombination.

^a^ In family10-11, as the unbalanced embryos were the only reference, the whole chromosome haplotypes couldn’t be established.

^b^ The breakpoints were identified by microarray results, except chromosome 22 in family1.

^c^ The carrier’s brother was used as a reference.
